# Use of Remote Assessment Tools to Substitute Routine Outpatient Care: Scoping Review

**DOI:** 10.2196/65938

**Published:** 2025-03-04

**Authors:** Jayne Barclay, Clair Sullivan, Michael Beckmann, Graeme Mattison, Rebecca Runciman, Elizabeth Martin

**Affiliations:** 1 School of Medicine University of Queensland Brisbane Australia; 2 Centre for Health Services Research Faculty of Medicine The University of Queensland Brisbane Australia; 3 Clinical Informatics Director (Research) Metro North HHS Queensland Health Brisbane Australia; 4 Mater Misericordiae Ltd Brisbane Australia; 5 QLD Digital Health Centre University of Queensland Brisbane Australia; 6 Department of Respiratory Medicine The Mater Hospital Brisbane Brisbane Australia; 7 Wesley Research Institute Brisbane Australia

**Keywords:** remote assessment, remote patient monitoring, telemedicine, mHealth, applications, patient-reported outcome measures, self-reported, health care cost reduction, hybrid care models

## Abstract

**Background:**

The increasing global demand for health care, driven by demographic shifts, the rise of personalized medicine, and technological innovations necessitate novel approaches to health care delivery. Digital remote assessment tools have emerged as a promising solution, enabling hybrid care models that combine traditional and remote patient management. These tools support the quadruple aim of health care by enhancing the monitoring and evaluation of patient-reported data, thereby improving patient care, boosting operational efficiency, reducing costs, and improving the experience of patients and clinicians. This review seeks to understand how remote assessment tools are used for routine consultation substitution in adult tertiary care centers.

**Objective:**

This scoping review aims to evaluate the implementation and health outcomes of digital remote assessment tools used for routine consultation substitutions in adult tertiary care centers. The objectives include assessing the extent of use, types, and effectiveness of these tools in substituting conventional outpatient care.

**Methods:**

A comprehensive scoping review was conducted, adhering to the PRISMA-ScR (Preferred Reporting Items for Systematic Reviews and Meta-Analyses extension for Scoping Reviews) checklist. The review focused on studies that used internet-dependent remote assessment technologies for patient data transfer in tertiary care settings. A detailed search strategy was used across multiple databases, with studies selected based on predefined inclusion and exclusion criteria. Data extraction and analysis were performed by independent reviewers, with a focus on the functionalities of the tools and their alignment with the Quadruple Aim of Healthcare.

**Results:**

The review included 12 studies, highlighting a growing interest in remote assessment technologies across diverse clinical settings. The interventions varied in length, from 4 weeks to 12 months, and demonstrated a range of functionalities, including symptom monitoring and postsurgical follow-ups. The use of these tools was associated with improved clinical outcomes, such as timely intervention for clinical deterioration and enhanced clinical protocol adherence. Additionally, a small number of studies identified potential cost savings in terms of reduced unplanned health care contacts and optimized clinical resource use. Patient and clinician experiences were generally positive, with high adherence to remote assessments and an appreciation for the personalized and timely care facilitated by these technologies. Barriers included high initial setup costs for digital technologies, leading to an inflated cost per patient in small sample studies.

**Conclusions:**

Digital remote assessment tools offer significant potential to enhance health care delivery by improving health outcomes, reducing costs, and enriching patient and clinician experiences. Their flexibility and adaptability make them suitable for various clinical contexts, supporting the personalization of care and operational efficiency. While digital remote assessment tools offer significant potential, careful consideration of implementation strategies, equity, cost, and clinician and patient experiences is crucial for successful clinical integration.

## Introduction

### Background

Globally, the demand for health care services is set to rise due to an increasing population, an aging demographic, the rise of personalized medicine, and the introduction of innovative technologies. Escalating costs, digital disruption, and consumer-centered care are likely to impact the effectiveness and viability of health care systems to meet this demand [1]. Novel approaches to health care are needed.

Well-established digital health interventions, such as telehealth have only marginally enhanced the capacity of public health systems and health care services [2]. Efforts to explore new strategies for enhancing health system sustainability persist, including digital interventions that use data to triage patients to optimal care pathways [3]. Remote management of diagnosed chronic diseases using biometric and wearable technologies is also becoming more common [3]. Such interventions streamline patient screening and triage, center the process around the patient, and in some instances, obviate the need for physical tertiary facility visits [4]. Provider-delivered remote monitoring tools and digital patient-reported outcome measurement tools are becoming prevalent. These approaches enable the introduction of new care models that may alleviate operational health system pressures and the realization of personalized care models [5].

Digital remote assessment tools are an emerging area with the potential to revolutionize traditional patient care approaches. These technologies enable hybrid care delivery models consisting of asynchronous patient-reported follow-up outside of conventional care settings and enable health providers to clinically supervise remotely located patients [5]. Such tools serve to capture, monitor, evaluate, and guide care decisions undertaken by health care professionals using the data reported by the patient, including pain, function, and symptoms.

Remote assessment tools cover a broad range of methods and technologies that can deliver interactive, patient-provider communications remotely through the digital collection of clinical assessment data. The implementation of care substitution using technology could yield benefits for both patients and providers by reducing unplanned health care contact post surgery, decreasing travel and costs, and optimizing workflows and human resources [6,7]. There is also some evidence that substituting postoperative follow-up visits with clinical assessment data collected from smartphone apps is acceptable to patients [6,7]. The broad use of “care substitution” encompasses various technologies that facilitate patient-provider interactions through the digital collection of clinical assessment data, thereby offering an alternative to traditional face-to-face or videoconferencing consultation.

The Quadruple Aim of Healthcare [8] provides a comprehensive framework for evaluating new interventions by focusing on 4 key dimensions: improving population health, enhancing patient and clinician experiences, and reducing health care costs, offering a holistic measure of an intervention’s impact and effectiveness. This general approach is particularly valuable in assessing diverse interventions, as it captures a wide range of outcomes and benefits, ensuring a thorough evaluation despite the variability in study designs and methodologies. The integration of innovative technologies to complement or substitute existing clinical pathways warrants further exploration, particularly the use of asynchronous remote consultations to replace routine tertiary facility care. There are no published systematic or broad scoping reviews that assess the outcomes of using only remote assessment tools to substitute routine outpatient care.

This review seeks to understand, through the available literature: How are adult patient interventions using remote assessment tools used, practically, and effectively, for routine consultation substitution in tertiary care centers?

### Study Objectives

The objectives of this scoping review are to (1) investigate how many studies have used remote assessment tools as the primary data collection or assessment method for routine care substitution; (2) explore what are the types, frequencies, and durations of interventions using digital assessment tools; and (3) map the published outcomes of interventions using digital assessment tools, including how they are evaluated and measured against the quadruple aim framework. This research can inform health care systems and providers regarding their appropriate use.

## Methods

### Overview

A scoping review was conducted, following PRISMA-ScR (Preferred Reporting Items for Systematic reviews and Meta-Analyses extension for Scoping Reviews) methods [9], because of the broad research questions and the emerging field of digital remote assessments. The PRISMA-ScR checklist, provided in Multimedia Appendix 1, was used to ensure comprehensive reporting and methodological rigor.

### Study Selection and Search Strategy

The intervention of interest for this scoping review was remote assessment tools using digital internet technologies incorporating patient-reported data in a tertiary health setting. The full inclusion and exclusion criterion developed is shown in Textbox 1.

The initial literature search was conducted in April 2023. An updated search was completed in March 2024. The search terms used to describe the intervention were “survey,” “telemedicine,” “mobile applications,” and “tertiary” with synonyms built out from these initial terms. Studies published in the last 10 years in English and indexed in the following databases were searched: PubMed, CINAHL, Embase, Web of Science, and Cochrane. The PubMed search strategy is included in Multimedia Appendix 2.

**Search strategy inclusion and exclusion criteria.**

**Inclusion criteria**

- Study type: Peer-reviewed, full-length studies.
- Language: English
- Study design: Primary intervention studies with or without a control group, including randomized controlled trials, nonrandomized controlled studies, controlled before-after studies, observational cohort studies, and preliminary and pilot studies.
- Population: Study participants aged 18 years or older, who are under management of a hospital or similar tertiary facility.
- Intervention: Remote assessments using internet-dependent technologies to transfer patient-reported data from the patient’s home to a hospital institution or tertiary health provider as the primary intervention.
- Comparison: Health facility or hospital where remote patient assessments were compared to standard or routine care, specifically face-to-face or in-person clinical consultations
- Outcome: Hospitalization rate, mortality, quality of life, health service efficiency, economic impact, clinical workflows, patient experience, clinician experience, and access to health services.

**Exclusion criteria**

- Study type: Systematic reviews, scoping reviews, study protocols, conference abstracts, case studies, or editorials or commentary.
- Language: Languages other than English.
- Study design: Studies without primary data, descriptive studies only, theoretical frameworks, conference abstracts, or posters.
- Population: Pediatric patients and patients managed outside of a tertiary or hospital team setting.
- Intervention: Exclude video consultations and technologies that directly transfer data from biometric or wearable devices; exclude the use of photographic data; and exclude studies that use educational information or content.
- Comparison: Studies assessing rehabilitation, exercise, mobility, or clinical education use and adoption compared to in-person consultations.
- Outcome: Educational outcomes and training effectiveness

All identified citations were uploaded into EndNote (version 20.6/2023; Clarivate Analytics) and initial duplicates were removed. The remaining studies were then imported into Covidence, a web-based literature review platform, where the identification of studies was undertaken by 3 independent reviewers. All 3 reviewers screened the titles and abstracts for eligibility, with any combination of 2 reviewers per study. A study was included if both reviewers agreed that it met the inclusion criteria. Conflicts in study selection were resolved during team meetings. Full-text review of studies was randomly distributed across the team of 3 reviewers, with any combination of 2 reviewers requiring agreement for the study to be included. Extraction of data from the final full-text studies was shared across all reviewers, with all reviewers systematically and independently documenting data as per a previously agreed data extraction format. The data extraction fields were then compared, discussed, and finalized with all reviewers present.

To help understand the functions of the tools used, the data was used to create a functionality score based on the Incidental Medical Services (IMS) Institute for Healthcare Informatics functionality score description [10]. This functionality score consists of 7 functionality criteria and 4 functional subcategories. The complete structure of the IMS Institute for Healthcare Informatics functionality scoring can be found in Table 1. Each intervention was scored using 1 to indicate function was present, and 0 function was not present.

To facilitate consistency across the studies, the results were mapped across the 4 dimensions of the Quadruple Aim of Healthcare to assist in the comparison of benefits and outcomes. The quadrants used were (1) improve population health, (2) enhance patient experience, (3) enhance clinician experience, and (4) reduce health system costs. In the context of this review, factors contributing to the reduction of health system costs under investigation included cost of care or service (overall operational cost to provide a service), workforce use, and reduction in avoidable or unnecessary hospital admissions.

**Table 1:** IMS^a^ Institute for Healthcare Informatics functionality criteria assessment [10].

Functionality criteria		Description
Inform		Provides information in a variety of formats (text, photo, and video)
Instruct		Provides instructions to the user
**Record**		Capture user entered data
	Collect data	Able to enter and store health data
	Share data	Able to transmit health data
	Evaluate data	Able to evaluate the entered health data by patient and provider, provider and administrator, or patient and caregiver
	Intervene	Able to send alerts based on the data collected or propose behavioral intervention or changes
Display		Graphically display user entered data or output user–entered data
Guide		Provide guidance based on user entered information, and may further offer a diagnosis, or recommend a consultation with a physician or a course of treatment
Remind or alert		Provides reminders to the user
Communicate		Provide communication with health care provider or provide links to social network

^a^IMS: Incidental Medical Services.

## Results

### Overview

**Figure 1:**
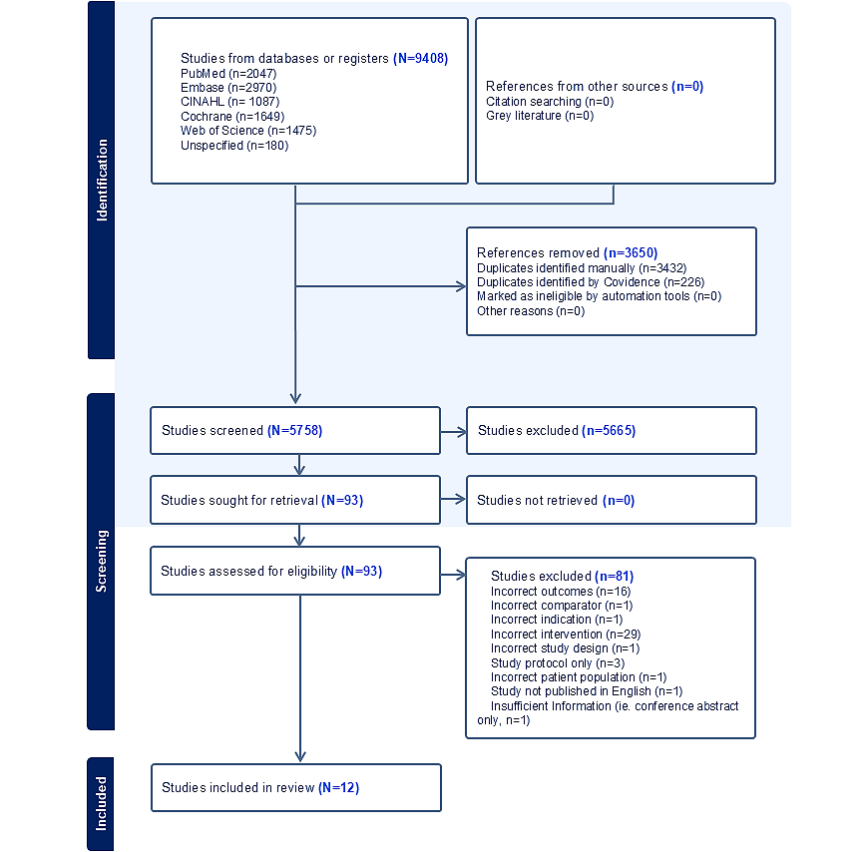
PRISMA (Preferred Reporting Items for Systematic Reviews and Meta-Analyses) flow diagram for combined searches.

The literature searches yielded 5758 unique citations, of which 12 studies were included in the final review. Figure 1 demonstrates the combined search results, with the first search completed in April 2023, and an updated search completed in March 2024.

### Summary of Included Studies

The 12 included studies span a publication period from June 2020 to January 2024, illustrating a growing contemporary interest in the domain of digital remote assessments. Geographically, the research landscape is diverse with studies originating from regions including the United Kingdom [11-13], Europe [13-17], North America [18,19], Asia [20], and Africa [21]. As for clinical focus, the studies encompass a wide range of patient populations, including those undergoing cancer treatment, individuals with cardiac or rheumatoid conditions, and postsurgery patients. Five of the studies included specific participant criteria that included participants who communicated in a specific or native language [12,15-17,19].

Intervention descriptions are varied, from real-time digital monitoring systems to smartphone apps. While some studies use the term “remote symptom monitoring” explicitly, others refer to “digital health tool,” “mHealth,” or “connected monitoring.” It should be noted that there were a considerable number of conference extracts that were excluded due to insufficient information published. Characteristics of included studies are presented in Multimedia Appendix 3 [11-22].

### Types of Remote Assessment Interventions

Observations from the results on interventions involving remote assessment tools in various clinical scenarios reveal certain care models and distinctions. The length of interventions varied across studies, ranging from a period of 4 weeks [14] to a more extended duration of up to 12 months [20], with others such as Sanyal et al [18] remaining unspecified.

### Data Sampling

Some studies adopted a weekly sampling approach [13,14,22], while others used more intermittent sampling, evident in Liu et al's method of sampling [20] at 90, 180, and 365 days. Richards et al [12] introduced a mixed approach, beginning with thrice weekly visits in the initial week and then weekly assessments to the eighth week. The assessments’ purpose ranged from symptom monitoring, and feedback collection to postsurgical outcomes and patient experience, with the data collection formats and tools varying accordingly. This included data collected through specific clinical symptom questionnaires designed by local clinical teams or disease-specific assessments, pain scores, patient experience Likert measures, distress thermometers, and disease-specific patient-reported outcome measures in various combinations.

### Technology

**Figure 2:**
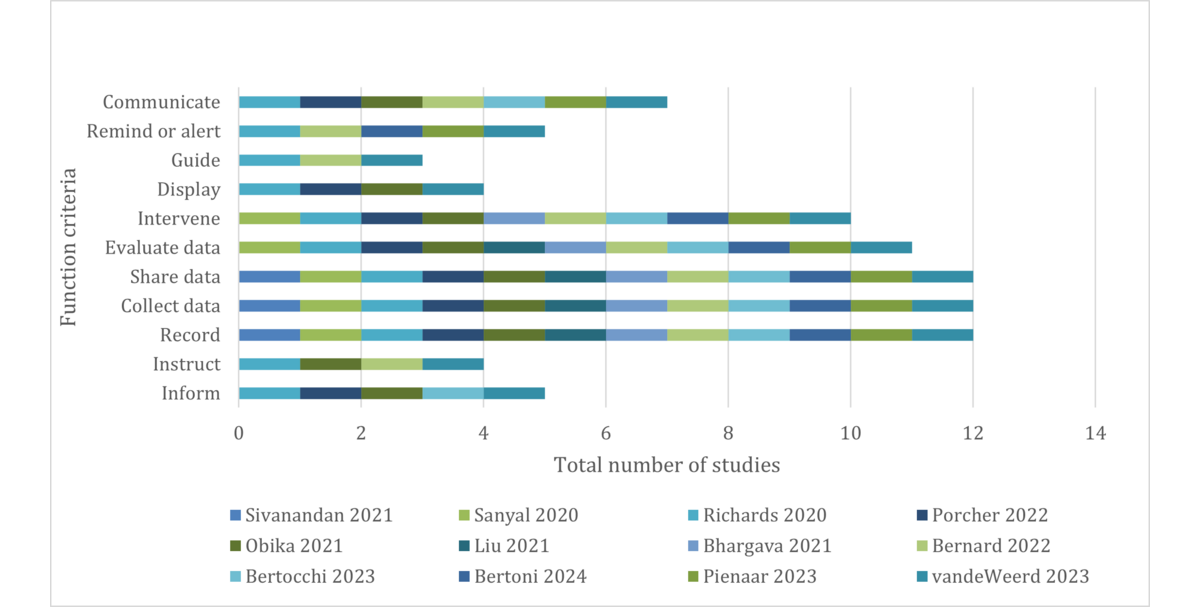
Study intervention assessed by digital health function criteria [11-22].

Interventions leveraged digital platforms, either on computers or smartphone devices. Several studies used digital, web-based questionnaire portals [11,19], with other studies [18,22] using smartphone-native apps, some of which had additional functionalities like real-time alerts for clinical teams based on patient input. Pienaar et al [21] use a “2-way-texting” platform that requires only native SMS text responses from patients. Functional variation of the technology used is described in Figure 2, mapped against the IMS Institute for Healthcare Informatics mHealth functionality criteria [10]. Only 1 study [18] incorporated a dual approach using both SMS and email to communicate with patients. The majority of interventions [11-19,22] used functionalities to trigger specific clinical team alerts or interventions when customized thresholds in symptom reports were reached. Only the functions of recording, collecting, and sharing data were consistent across all interventions reviewed.

### Evaluation of Outcomes Mapped to the Quadruple Aim

With an extensive range of variation across the types of interventions reviewed, there was not a single or consistent evaluation method to measure the outcomes and benefits of the intervention. The themes of the included studies are summarized in Multimedia Appendix 4 [11-22].

### Population Health Outcomes

Overall, the use of remote assessment tools to directly manage patient health outcomes indicated positive results, particularly in the management of clinical deterioration, timely clinical intervention, and clinical protocol adherence. Accurate identification of clinical deterioration was achieved in most studies [12-14,19,22], whereby remote clinical review was initiated after interpretation or alert of the data, and patient treatment schedules or pathways were adjusted to prevent harm or further deterioration of health. This included 1 study that reported no patients on the intervention attending an emergency department for symptom management during the study period [19] and a reduction of the 30-day readmission rate for patients with high protocol adherence in a separate study [15]. In another study, quality of life outcomes (quality-adjusted life years) improved by 0.07, on a scale of 0 to 1, for those patients who underwent remote digital assessment as compared to traditional in-person monitoring and consultations [13]. The number of potential adverse events was also tracked over the intervention period in 1 study, where potential adverse events were low on day 1 post surgery and peaking on day 3 of 13-day monitoring [21].

One study explored the consistency of patients’ data provided over a telephone interview with a clinician versus patient self-reporting via instant messaging. The results showed up to 10% variance on some symptom assessment data points, which may impact accurate management of health outcomes [20].

### Health System Costs

#### Cost of Care or Service

Two studies [13,19] attempted to understand service costs using cost utility analysis, assessing the overall cost saving of providing care through remote assessment interventions compared to usual care. While both studies reported a cost saving delivered by the intervention compared to routine care, there was commentary on an inflated cost per patient due to the small sample sizes undertaken, n=13 [19] and n=89 [13], and the baseline costs of establishing the digital technologies for such interventions. The majority of patients in 1 (60%) study indicated that the digital intervention saved them time and money from attending in-person clinic visits [21].

#### Workforce Use

The impact on the use of clinical resources through the substitution of remote assessment was reported across several studies [12-14,19,20], including the assumed suggestion of potential cost savings for the health service through triaged use of multidisciplinary teams, asynchronous communication, and reduction of routine consultation methods. The ability to effectively use different roles where appropriate was highly dependent on predefined patient pathways and parameters that could be automated through the digital tools and workflows used [12-14,19,22].

#### Reduction in Avoidable or Unnecessary Hospital Admissions

There was no clear reporting on the reduction in avoidable or unnecessary hospital admissions. The number of hospital readmissions for the intervention group of postsurgical patients was reported, however, no comparator to readmissions for a usual care approach was provided to establish if this was an improvement or otherwise [12]. One study reported a correlation of reduction in readmission rate for patients with high adherence to the digital protocol (*P*=.004) [15]. A reduction in hospitalization rate over a 6-month period was reported in patients with chronic disease when remotely monitored (rate of hospitalization=1.3) compared to the conventional monitoring group (rate of hospitalization=2.3) [13].

### Patient Experience

Patient-experience measures were commonly captured through the use and adherence of the protocol, as well as qualitative surveying of the patient’s service experience using Likert scoring.

Overall, there was positive patient acceptance of services using digital remote assessments [13-16,19,21,22], with adherence levels to the assessment reporting ranging between 62%-93% across the full study periods of the relevant interventions.

One study noted that patients with high function and low symptom burden were more likely to withdraw, implying the tool was more beneficial for patients experiencing significant symptoms [19]. It was further noted that there seemed to be other influencing factors to patient use such as physical health, in being able to complete self-reporting assessments [19].

Further thematic analysis of 1 intervention [12] highlighted that most patients valued the advice provided by the technology-enabled system. Key themes included reassurance and the relevance of advice which felt tailored to their specific needs. Patient perception of the usefulness of the interventions varied, with 1 study [17] reporting that 63% of patients thought the intervention was useful to support the management of health and access to services (score of 4.42/7), while another scored above 92% for patients feeling comfortable and safe using the digital service [21].

### Clinician Experience

The 2 study interventions where clinical experience measures were reported both strongly indicate positive outcomes [12,19]. Measures indicated a consensus among clinical staff that using digital assessments improved clinical confidence in the delivery of care. Commentary in these studies indicated clinicians’ belief that no clinical symptoms were overlooked, and the system was perceived to provide additional safety in highlighting areas of patient health that might not otherwise be reported or escalated by the patient [12,19].

One study highlighted that the clinicians found the ability to access the reported data through the hospitals. Electronic medical record systems useful, however, were contingent upon their accessibility to a computer within the hospital [12].

Additionally, 75% of clinicians from 1 study [19] perceived that the quality of life for their patients had improved owing to the remote assessment system in place.

## Discussion

### Summary of Evidence

This scoping review seeks to explore how remote assessment tools are used, practically and effectively, for routine consultation substitution in tertiary care centers. The review identifies the risks and benefits of these tools to guide health care systems in adopting technology-assisted models of care. Specifically, it investigates studies using remote assessment tools as the primary method for routine care substitution, the types and durations of these interventions, and how published outcomes align with the Quadruple Aim framework.

The review identified 12 studies, indicating that while digital remote assessment tools may be broadly implemented across health care facilities, limited research has been published on their specific applications for routine care substitution. Despite this small body of literature, these tools appear to be effective in capturing and assessing clinical deterioration, enabling proactive management and personalized treatment pathways. They may influence care pathway stratification and multidisciplinary team use and have the potential to improve health service efficiency.

The geographic distribution of studies suggests a concentrated focus in regions such as the United Kingdom and Europe, where conditions appear favorable for this type of research. However, many studies excluded participants due to language barriers, raising concerns about equity and inclusivity. While digital translation tools are widely available in other industries, health care settings face unique challenges in integrating such solutions effectively [23].

Notably, all studies were published from 2020 onward, highlighting the role of the COVID-19 pandemic as a catalyst for the adoption and evaluation of digital remote assessment tools. This trend underscores the growing interest in leveraging digital health technologies to address emerging pressures on health care systems. However, the limited number of studies underscores the need for further research to establish robust evidence for the risks, benefits, and broader applicability of these interventions.

### Principal Findings

When analyzed using the Quadruple Aim framework, the findings demonstrate an emphasis on measuring population health outcomes and patient experience. However, the neglect of health system costs and clinician experience in many studies reveals a significant gap in literature. To create a sustainable health care system capable of meeting both current and future challenges, balanced attention to all 4 dimensions of the Quadruple Aim is essential [8].

Only 2 studies [13,19] examined health system costs, underscoring the limited focus on this critical area. This neglect may stem from the challenges associated with costing health care delivery comprehensively, particularly in complex, innovative care models like digital health interventions. Traditional costing methods often fail to capture the variability and complexity of care pathways, making it difficult for policymakers to evaluate economic implications accurately [24]. Without robust cost analyses, the ability to optimize resource allocation, reduce unnecessary expenditures, and understand long-term sustainability remains limited [25].

Similarly, only 2 studies [12,19] addressed clinician experience, a surprising finding given the pivotal role clinicians play in the success of digital health technologies. While the focus on patient-centered care is appropriate, neglecting clinician perspectives risks compromising the balance necessary to achieve the Quadruple Aim [26]. Clinicians must be confident that digital remote assessment tools enhance, rather than burden, their workflows and align with their commitment to patient care. Failure to address these concerns could lead to implementation challenges or resistance, undermining the benefits of digital interventions.

### Limitations

The study has several limitations. The narrow selection criteria focused exclusively on interventions using remote assessment data as the primary data source, excluding studies that integrated additional data inputs such as biomedical devices, photographic data, or web-based education. As a result, relevant studies incorporating more comprehensive data sources were not included.

Additionally, a substantial number of studies (n=27) were excluded due to insufficient information to answer the research questions, many of which were conference abstracts. The lack of randomized controlled trials or comparator studies further limits the robustness of the findings, potentially introducing bias and inflating the perceived benefits of these interventions. This reflects a broader challenge in digital health research, where the rapid pace of technological development renders traditional randomized controlled trials less feasible. Innovative methods, such as observational studies, may provide more pragmatic and timely approaches for evaluating digital health interventions [27].

### Future Research

Future research should prioritize direct assessments of health system costs associated with remote assessment interventions, incorporating time-driven activity-based costing to provide more accurate and comprehensive insights [25]. Understanding funding policies and potential revenue streams from public and private health funders will also be essential to support scalability and sustainability [27-29].

Additionally, research should explore clinician experiences with reduced patient contact due to digital interventions and identify systemic strategies to mitigate burnout and promote health care workers’ well-being. This area remains underexplored despite its importance for successful implementation.

### Broader Implications

The integration of digital remote assessment tools into health systems holds substantial promise for improving health care delivery. However, their success hinges on addressing critical challenges, including equity, cost analysis, and clinician engagement. Tailoring interventions to specific clinical contexts, integrating them seamlessly with existing workflows, and incorporating patient and clinician feedback are essential steps toward maximizing their impact.

To ensure long-term sustainability, future research and implementation efforts must adopt a holistic approach that balances the dimensions of the Quadruple Aim. This includes developing robust cost analyses, fostering clinician buy-in, and addressing barriers to accessibility. Ultimately, by addressing these factors, digital health interventions can contribute to a more equitable, efficient, and patient-centered health care system capable of withstanding evolving challenges.

### Conclusions

This review highlights the broad-reaching potential and adaptability of digital remote assessment interventions. Despite variability in duration and methodologies, these technologies consistently demonstrate accurate data recording, collection, and dissemination. More mature interventions that guide clinical responses offer additional benefits.

Digital health solutions can advance the Quadruple Aim of Healthcare by enhancing population health outcomes, optimizing resource allocation, and improving patient and clinician experiences. Future research should establish standardized measures of health system effectiveness, consider clinician adoption, and investigate the long-term sustainability and impact on access to care.

While digital remote assessment tools offer significant potential, careful consideration of implementation strategies, equity, cost, and clinician and patient experiences is crucial for successful clinical integration.

## Data Availability

The PRISMA-ScR (Preferred Reporting Items for Systematic Reviews and Meta-Analyses extension for Scoping Reviews) checklist was included in the manuscript submission and can be consulted in Multimedia Appendix 1. The datasets generated and analyzed during this study are available from the corresponding author (JB) on reasonable request.

## Appendix

Multimedia Appendix 1 PRISMA (Preferred Reporting Items for Systematic Reviews and Meta-Analyses) checklist for scoping review study design.

Multimedia Appendix 2 PubMed search strategy used to undertake scoping review.

Multimedia Appendix 3 Summary of study characteristics.

Multimedia Appendix 4 Summary of study themes mapped across the Quadruple Aims of Healthcare.

## Abbreviations

IMS: Incidental Medical Services
PRISMA: Preferred Reporting Items for Systematic Reviews and Meta-Analyses
PRISMA-ScR: Preferred Reporting Items for Systematic Reviews and Meta-Analyses extension for Scoping Reviews
