# Supplementary material for: Use of Remote Assessment Tools to Substitute Routine Outpatient Care: Scoping Review
Source: J Med Internet Res. 2025 Mar 4;27:e65938. doi: 10.2196/65938 (PMC11920651; doi:10.2196/65938)
Supplement: Multimedia Appendix 2 [file jmir_v27i1e65938_app2.docx]

**PubMed Search Strategy**

"Surveys and Questionnaires"[MeSH Terms] OR "Patient Reported Outcome Measures"[MeSH Terms] OR "survey*"[Title/Abstract] OR "questionnaire*"[Title/Abstract] OR "symptom assessment*"[Title/Abstract] OR "patient reported outcome*"[Title/Abstract] OR "PRO2"[Title/Abstract] OR "patient symptom*"[Title/Abstract] OR "patient reported symptom*"[Title/Abstract] OR "patient assessment*"[Title/Abstract] OR "patient generated data"[Title/Abstract] OR "patient monitor*"[Title/Abstract] OR "symptom monitor*"[Title/Abstract] OR "patient reported symptom*"[Title/Abstract] OR "patient assessment*"[Title/Abstract] OR “self-management”[Title/Abstract] OR “self management”[Title/Abstract] OR “self report”[Title/Abstract] OR “self-report”[Title/Abstract]

AND

"Telemedicine"[MeSH Terms] OR "remote online"[Title/Abstract] OR "remote monitor*"[Title/Abstract] OR "Telemedicine"[Title/Abstract] OR "telemonitor*"[Title/Abstract] OR "remote follow-up"[Title/Abstract] OR "remote consultation*"[Title/Abstract] OR "asynchronous consultation*"[Title/Abstract] OR home[Title/Abstract] OR "mHealth"[Title/Abstract] OR "m-health"[Title/Abstract] OR "mobile health"[Title/Abstract] OR telehealth[Title/Abstract] OR “virtual medicine”[Title/Abstract] OR “virtual health”[Title/Abstract] OR “asynchronous consult*”[Title/Abstract] OR “real-time support”[Title/Abstract]

AND

Mobile Applications[MeSH Terms] OR "mobile health platform*"[Title/Abstract] OR "digital intervention*"[Title/Abstract] OR "smartphone*"[Title/Abstract] OR "mobile*"[Title/Abstract] OR "iphone*"[Title/Abstract] OR "app"[Title/Abstract] OR "apps"[Title/Abstract] OR "internet*”[Title/Abstract] OR "mobile application*"[Title/Abstract] OR "electronic assessment tool*"[Title/Abstract] OR "electronic survey*"[Title/Abstract] OR "mobile application*"[Title/Abstract] OR "portable software*"[Title/Abstract]

AND

"Tertiary"[Title/Abstract] OR "hospital*"[Title/Abstract] OR "post discharge"[Title/Abstract] OR "post surgery"[Title/Abstract] OR “After care” [Title/Abstract] OR “post-operative” [Title/Abstract] OR “patient discharge” [Title/Abstract] OR “post acute” [Title/Abstract] OR “post-acute” [Title/Abstract] OR "Aftercare"[Mesh]
